# 5 year incidence of YAG capsulotomy and PCO after cataract surgery with single-piece monofocal intraocular lenses: a real-world evidence study of 20,763 eyes

**DOI:** 10.1038/s41433-019-0630-9

**Published:** 2019-10-15

**Authors:** Paul G. Ursell, Mukesh Dhariwal, Derek O’Boyle, Javeed Khan, Alessandra Venerus

**Affiliations:** 1 Epsom & St Helier University Hospitals NHS Trust, Epsom, Surrey, UK; 2 Alcon Vision LLC, Fort Worth, TX USA; 3 Alcon Laboratories Ireland Ltd., Cork, Ireland; 4 IQVIA, London, UK

**Keywords:** Risk factors, Health care economics, Outcomes research

## Abstract

**Objectives:**

To evaluate the 3- and 5-year incidence of posterior capsule opacification (PCO) and neodymium-doped yttrium aluminium garnet (Nd:YAG) capsulotomy in patients following cataract surgery, comparing results for different single-piece acrylic hydrophilic and hydrophobic monofocal intraocular lens (IOL) models and other patient factors.

**Patients and methods:**

Electronic medical record data collected from seven United Kingdom (UK) National Health Service (NHS) ophthalmology clinics for routine, age-related (≥65 years) cataract surgeries that implanted single-piece acrylic monofocal IOLs during 2010–2013 were used to calculate 3- and 5-year incidence of Nd:YAG and PCO. IOL models of Alcon Acrysof, AMO Tecnis, Bausch & Lomb (B & L) Akreos, LenStec Softec, and Rayner Flex were analyzed. Pairwise comparisons were conducted between AcrySof IOLs and other IOLs using Bonferroni adjustment for multiplicity. Multivariate analyses were conducted adjusting for known confounders.

**Results:**

The incidence of Nd:YAG capsulotomy ranged between 2.4–12.6% at 3 years and 5.8–19.3% at 5 years post-cataract surgery. Similarly, the incidence of PCO ranged between 4.7–18.6% at 3 years and 7.1–22.6% at 5 years. When comparing all of the single-piece IOLs, AcrySof demonstrated the lowest incidence rates for both PCO and Nd:YAG (*P* < 0.001 for each comparison). From adjusted logistic regression analysis, AcrySof were associated with lower 3- and 5-year odds of Nd:YAG and PCO incidence.

**Conclusions:**

Following cataract surgery with single-piece monofocal IOLs different incidence rates of PCO were observed with different IOLs. AcrySof IOLs were associated with significantly lower incidence of PCO requiring Nd:YAG treatment over periods of 3 and 5 years.

## Introduction

Posterior capsule opacification (PCO) is the most frequent complication of cataract surgery and can develop soon after to a few years post-procedure [1, 2], with incidence figures ranging from <5% to as high as 50% [3]. PCO involves lens epithelial cell growth and proliferation, leading reduced visual acuity, and may develop in a few months to years following cataract surgery [4, 5].

Neodymium-doped yttrium aluminium garnet (Nd:YAG) laser capsulotomy is the only effective surgical treatment for PCO [4], and is a routine and largely safe procedure, but could be associated with occasional complications that include elevated intraocular pressure, retinal detachment, and endophthalmitis [4–6]. The requirement to perform Nd:YAG capsulotomies as a consequence of PCO places a considerable financial burden on healthcare systems. This is due to the costs of the procedure itself, follow-up visits, and also managing the associated complications that may arise as a result of the procedure [1, 2, 6].

The risk of PCO is understood to be influenced by a number of factors, including edge design, IOL design, haptic design [5], and lens material [7]. While there are known risk factors (such as round vs. sharp edge), more research is needed to understand the role of intraocular lens material in PCO risk [8]. Previous studies evaluating the incidence of Nd:YAG capsulotomy in patients with different IOL types suggest that more favourable outcomes have been shown for hydrophobic acrylic lenses compared with those made from other materials, including silicone and hydrophilic acrylic IOLs [9–13].

In long-term observational studies (3–9 years post-cataract surgery) looking at the incidence of both PCO and Nd:YAG following cataract surgery, hydrophobic acrylic IOLs have been associated with a longer time until the need for Nd:YAG capsulotomy, with less frequent [9, 11, 14] and less severe [11] or dense [14] PCO, and with lower per-patient post-operative costs [6].

The current study is an extension and post-hoc analysis of a retrospective, real-world evidence cohort study, which examined the impact of IOL biomaterial on PCO and Nd:YAG capsulotomy at 3 years post-cataract surgery and compared AcrySof IOLs with cohorts of other hydrophobic and hydrophilic IOLs [8]. The objective of this current analysis was to evaluate the long-term incidences of Nd:YAG and PCO in patients following cataract surgery at 3 and 5 years, comparing results for single-piece acrylic IOLs; and to evaluate 3- and 5-year Nd:YAG and PCO odds ratios based on IOL model and other covariates. Extending the previously published cohort analysis to individual IOL group level analysis provided an opportunity to compare the Nd:YAG incidence for each IOL, to inform clinical decision making, considering the fact that IOL material or design may affect post-surgery outcomes [7].

Longitudinal real-world evidence studies can supplement clinical insights gained from randomized clinical trials by providing robust long-term outcomes for large cohorts of patients that reflect routine clinical practice and account for real-world conditions, such as multiple patient comorbidities [15, 16]. Bodies such as the FDA (USA) and NICE (UK) are increasingly relying on real-world evidence to support healthcare decision making [16, 17]. In this analysis, PCO and Nd:YAG incidence were evaluated in various single-piece acrylic IOLs as an extension to the results reported in the 3-year cohort analysis [8].

## Materials and methods

### Data source

As described in the first publication [8], this study was designed as a longitudinal, retrospective cohort study. We sourced electronic medical records (EMRs) data collected from seven UK ophthalmology clinics for routine, age-related (≥65 years) cataract surgeries 2010–2013, and calculated the 3- and 5-year incidence of Nd:YAG capsulotomy and PCO in eyes implanted with acrylic monofocal IOLs over the follow-up period of 2010–2016. Among this overall study population, single-piece IOLs represented 92% of the total sample therefore in the present study, we analyzed this largest subgroup (>90% of eyes) of single-piece IOL models. Patients who died within the (3-year or 5-year) follow-up period were excluded from the 3-year and 5-year analyses, respectively. Table 1 describes the study inclusion and exclusion criteria, and the resulting study population. The National Health Service (NHS) sites for this study were selected on the basis of large numbers of procedures, reliable recording of cataract surgeries and post-operative follow-up data, including records of Nd:YAG capsulotomy and recording of PCO in the Medisoft EMR system. The site selection criteria on usage of Medisoft EMR system was justified because it is a widely used and validated [18, 19] EMR system covering >50% of NHS, UK cataract clinics, and because the data is highly structured and has the possibility to track patients longitudinally.

**Table 1:** Attrition table based on inclusion/exclusion criteria

Inclusion criterion	Eyes		Patients	
	*n*	%	*n*	%
Total patients records extracted			178,281	
Total patients with a record of cataract surgery with single piece, non-toric, monofocal, acrylic lenses with at least 500 appearances	160,995	100.0%	114,740	100.0%
Details on monofocal IOL implanted in surgery	160,685	99.8%	114,588	99.9%
Age ≥65 years on the date of first cataract surgery	136,911	85.0%	97,541	85.0%
Excluding capsular tension ring, vitrectomy, or posterior capsule rupture during cataract surgery	136,610	84.9%	97,415	84.9%
Excluding patients who had a record of “Vitrectomy”, “Previous pars plana vitrectomy”, or “PPV—not specified” as a copathology	135,686	84.3%	96,670	84.3%
Excluding eyes with >1 of any type (including minor surgeries) of surgeries (i.e. patients with >1 cataract on the same eye)	135,598	84.2%	96,622	84.2%
Eyes operated within the study period 2010–2013	48,527	30.1%	36,703	32.0%
Excluding eyes from patients who died within 3 years of their index date (i.e. during their follow-up)	47,754	29.7%	36,107	31.5%
Excluding eyes from patients who died within 5 years of their index date (i.e. during their follow-up) or who had cataract surgery earlier than 2011	20,763	12.9%	16,595	14.5%

### Study population

Eye-level data recorded between January 1, 2010 and December 31, 2016, was extracted for all eyes that had undergone cataract surgery in the selected clinics. Study inclusion criteria: eyes with a record of cataract surgery between January 1, 2010 and December 31, 2013, a record of in-the-bag placement of monofocal IOLs during cataract surgery, details on the type of IOL implanted, age ≥65 years, and *N* ≥ 500 for each IOL group to ensure sample parity between treatment groups. For the 5-year follow-up period, only eyes with cataract surgeries performed between 2010 and 2011 were included.

### Statistical analyses

The analysis was carried out in two steps. The first was a pairwise analysis, in which 3- and 5-year incidence of PCO and Nd:YAG capsulotomy were compared by IOL groups (reference group: AcrySof). Incidence proportion was calculated within each group IOL group as the total number of eyes developing the outcome, divided by the total eyes at risk in that group, adjusting therefore for the different groups’ size.

The Bonferroni method was used to adjust for multiplicity; four comparisons were carried out, therefore, each comparison was conducted at a level of significance of 0.0125.

In the second step, an adjusted logistic regression was performed to adjust for other known confounders besides the IOL model (reference group: AcrySof), including age at index, gender, number of eyes operated, intra- and post-cataract-surgery complications (e.g. hyphaema, hypotony, retained soft lens matter), presence of copathologies (e.g. diabetic retinopathy, glaucoma, high myopia), pupil size of the patient, incision site, seniority of surgeon, use of any trypan blue during the surgery, IOL power at index, and best distance-corrected visual acuity (BDVA) at index (per 1 logMAR increase). A stepwise selection process was used with a 5% level of significance for the covariates selection in the logistic model.

The two outcomes of interest were identified through information recorded in the patients’ clinical records; in particular, PCO was identified as any record including “Posterior Capsule Opacification”, while Nd:YAG was identified using OPCS code [20] associated with recorded procedures (OPCS-4 code C73.3 + Y08.6, indicating respectively “capsulotomy of posterior lens capsule” and “laser incision of organ”).

## Results

The IOLs contributing to this analysis are shown in Table 2.

**Table 2:** Intraocular lenses (IOLs) included in the analysis

IOL Group	Optic material	Composition	Eyes (*N*) (3-year analysis)	Eyes (*N*) (5-year analysis)
AMO Tecnis	Hydrophobic acrylic	Copolymer of ethylacrylate, ethyl methacrylate, 2,2,2-trifluroethyl methacrylate cross linked with ethylene glycol dimethacrylate phenylethyl acrylate and phenylethyl methacrylate cross linked with 1.4 butanediol diacrylate [31]	15,083	5609
Alcon AcrySof	Hydrophobic acrylic	Copolymer of phenylethyl acrylate and phenylethyl methacrylate cross linked with 1.4 butanediol diacrylate [31]	12,870	5342
B & L Akreos	Hydrophilic acrylic	Acrylic polymer^a^	9344	6847
Lenstec Softec	Hydrophilic acrylic	Hydroxyethylmethacrylate (HEMA, 26% water content) and a polymerizable UV blocker [32]	6274	2964
Rayner Flex	Hydrophilic acrylic	2 hydroxyethyl methacrylate/methyl methacrylate polymer [33]	4183	1

AMO Tecnis group consists of ZCB00; Alcon AcrySof group consists of SA60AT and SN60WF IQ; B & L Akreos group consists of Adapt and MICS MI60; Lenstec group consists of Softec 1 and Softec HD; Rayner Flex group consists of C-Flex 970C and Superflex 920H lenses

^a^IOL polymer name could not be retrieved from technical specification/other online resources

### 3- and 5-year incidence of Nd:YAG capsulotomy

Data from 47,754 eyes contributed to the 3-year follow-up analyses and data from 20,763 eyes contributed to 5-year follow-up analyses.

**Fig. 1:**
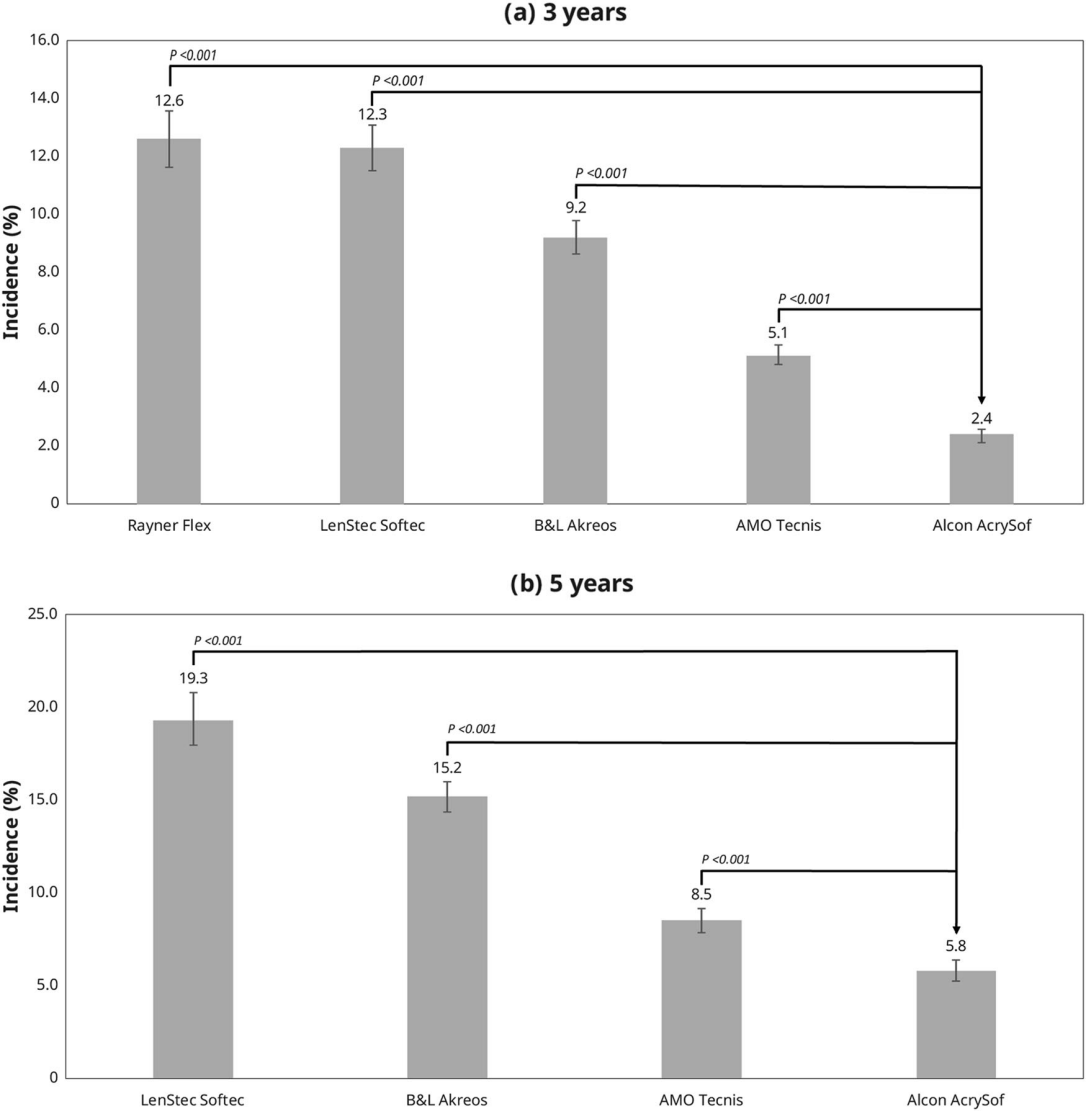
Incidence of Nd:YAG capsulotomy at 3 years (**a**) and 5 years (**b**)

During the study period, the 3-year incidence of Nd:YAG was: Alcon AcrySof (2.4% [307/12,870]; 95% CI: 2.1–2.6%), AMO Tecnis (5.1% [770/15,083]; 95% CI: 4.8–5.5%), B&L Akreos (9.2% [860/9344]; 95% CI: 8.6–9.8%), Lenstec Softec (12.3% [770/6274]; 95% CI: 11.5–13.1%), and Rayner Flex series (12.6% [526/4183]; 95% CI: 11.6–13.6%) (Fig. 1).

The 5-year incidence of Nd:YAG capsulotomy was: Alcon AcrySof (5.8% [309/5342]; 95% CI: 5.2–6.4%), AMO Tecnis (8.5% [477/5609]; 95% CI: 7.8–9.2%), B&L Akreos (15.2% [1038/6847]; 95% CI: 14.3–16.0%), and Lenstec Softec (19.3% [573/2964]; 95% CI: 17.9–20.8%) (Fig. 1).

### 3- and 5-year incidence of PCO

**Fig. 2:**
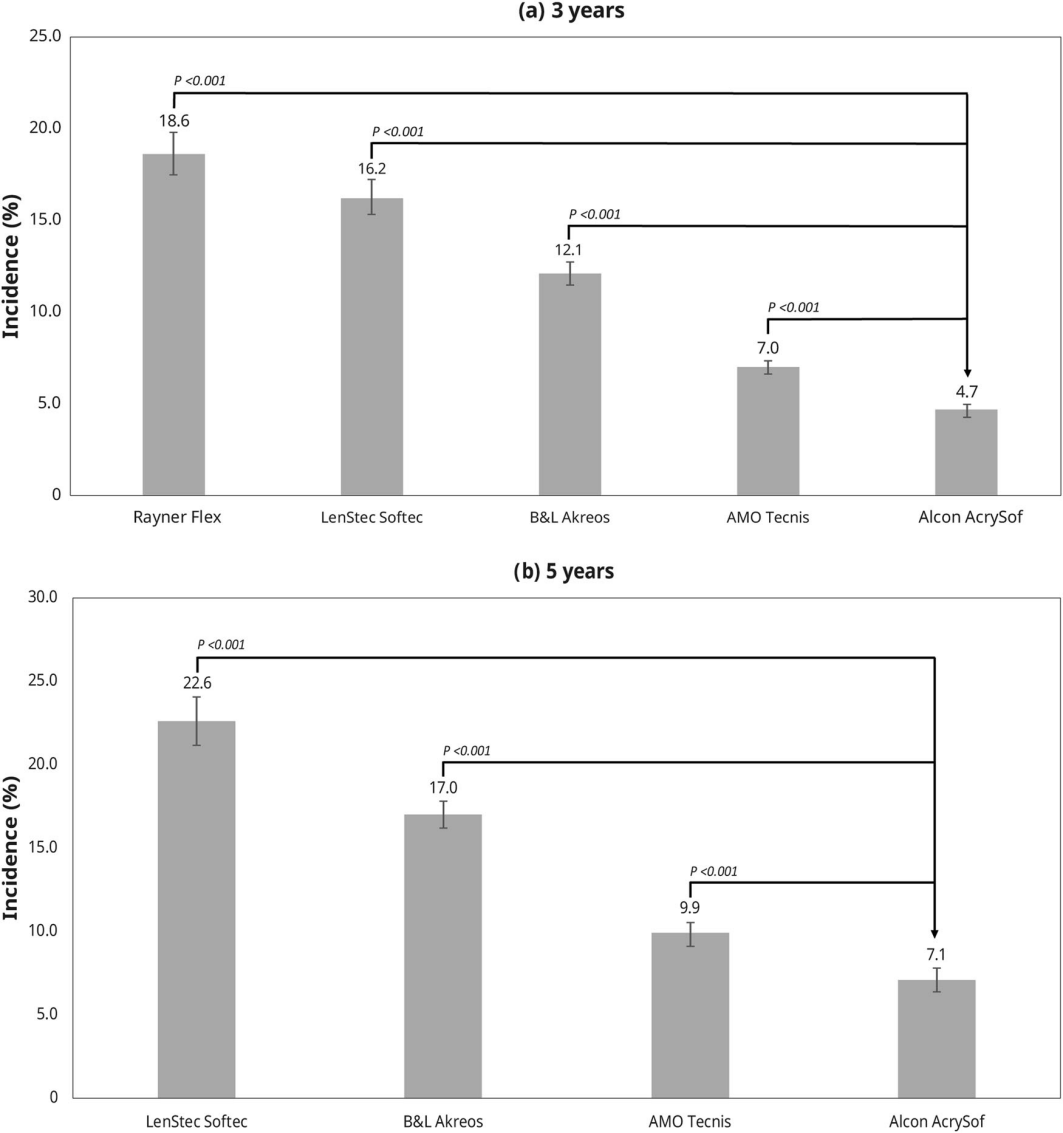
Incidence of PCO at 3 years (**a**) and 5 years (**b**)

The 3-year incidence of PCO was: Alcon AcrySof (4.7% [601/12,870]; 95% CI: 4.3–5.0%), AMO Tecnis (7.0% [1060/15,083]; 95% CI: 6.6–7.4%), B&L Akreos (12.1% [1135/9344]; 95% CI: 11.5–12.8%), Lenstec Softec (16.2% [1018/6274]; 95% CI: 15.3–17.1%), Rayner flex series (18.6% [777/4,183]; 95% CI: 17.4–19.8%) (Fig. 2).

The 5-year incidence of PCO was: Alcon AcrySof (7.1% [381/5342]; 95% CI: 6.4–7.8%), AMO Tecnis (9.9% [553/5609]; 95% CI: 9.1–10.6%), B&L Akreos (17.0% [1167/6847]; 95% CI: 16.2–17.9%), Lenstec Softec (22.6% [669/2964]; 95% CI: 21.1–24.1%) (Fig. 2).

### Adjusted logistical regression analysis

Odds ratios of Nd:YAG and PCO at 3 and 5 years by covariate (including lens type) and obtained from the logistic regression models are shown in Table 3. Similar to the unadjusted incidence calculations, the Alcon AcrySof IOLs were less likely to undergo Nd:YAG or PCO at 3 and 5 years compared with AMO Tecnis, B&L Akreos, Lenstec Softec, and Rayner Flex series IOLs (*P* < 0.001 for each comparison).

**Table 3:** Adjusted logistic regression analysis: Odds ratios of Nd:YAG and PCO at 3 and 5 years

Covariate	Sub-category	Comparator	OR (95% CI) for Nd:YAG		OR (95% CI) for PCO	
			3 years	5 years	3 years	5 years
IOL	AMO Tecnis	AcrySof	2.15^a^ (1.87–2.46)	1.46^a^ (1.25–1.70)	1.49^a^ (1.34–1.65)	1.36^a^ (1.18–1.57)
	B & L	AcrySof	4.25^a^ (3.71–4.88)	2.80^a^ (2.44–3.21)	2.95^a^ (2.65–3.28)	2.74^a^ (2.41–3.11)
	Lenstec	AcrySof	5.34^a^ (4.64–6.14)	3.83^a^ (3.29–4.45)	3.65^a^ (3.27–4.07)	3.52^a^ (3.06–4.06)
	Rayner	AcrySof	6.03^a^ (5.20–7.00)	0.00 (0.00–0.00)	4.73^a^ (4.21–5.32)	0.00 (0.00–0.00)
Age category	71–75 years	65–70 years	0.92 (0.81–1.05)	1.05 (0.90–1.23)	0.90 (0.81–1.01)	0.90 (0.78–1.04)
	76–80 years	65–70 years	0.98 (0.87–1.11)	1.08 (0.93–1.26)	0.91 (0.82–1.01)	0.99 (0.86–1.13)
	81–85 years	65–70 years	0.84^a^ (0.74–0.95)	0.92 (0.79–1.07)	0.85^a^ (0.77–0.95)	0.81^a^ (0.70–0.93)
	86–90 years	65–70 years	0.74^a^ (0.64–0.85)	0.72^a^ (0.61–0.86)	0.69^a^ (0.61–0.78)	0.61^a^ (0.52–0.72)
	≥90 years	65–70 years	0.48^a^ (0.38–0.60)	0.54^a^ (0.41–0.71)	0.47^a^ (0.38–0.57)	0.46^a^ (0.36–0.60)
Gender	Female	Male	1.16^a^ (1.08–1.26)	1.24^a^ (1.13–1.36)	1.18^a^ (1.11–1.26)	1.20^a^ (1.10–1.31)
Number of eyes operated	2	1	1.19^a^ (1.10–1.28)	1.29^a^ (1.18–1.41)	1.18^a^ (1.10–1.25)	1.32^a^ (1.22–1.44)
Post-operative complications	Yes	No	3.03^a^ (1.86–4.92)	4.76^a^ (2.65–8.53)	4.15^a^ (2.74-6.29)	4.50^a^ (2.53–8.01)
BDVA at index (per 1 logMAR increase)			0.71^a^ (0.62–0.82)	0.59^a^ (0.49–0.71)	0.87^a^ (0.77–0.98)	0.71^a^ (0.60–0.83)
IOL power at index (per 1-unit increase)			0.98^a^ (0.97–0.99)	0.97^a^ (0.96–0.99)	0.99^a^ (0.98–1.00)	0.98^a^ (0.97–0.99)
Surgeon seniority	Junior trainee	Consultant	0.95 (0.79–1.15)	NA	0.98 (0.84–1.15)	1.02 (0.82–1.27)
	Senior trainee	Consultant	0.84^a^ (0.77–0.92)	NA	0.85^a^ (0.78–0.91)	0.88^a^ (0.79–0.97)
Pupil size	Large	Small	NA	1.31 (0.99–1.72)	NA	1.11 (0.87–1.42)
	Medium	Small	NA	1.10 (0.81–1.51)	NA	0.91 (0.68–1.21)
Use of Vision Blue on date of cataract surgery	Yes	No	NA	NA	0.80^a^ (0.66–0.97)	0.76^a^ (0.59–0.99)
Copathologies recorded prior to or on index date	Yes	No	1.42^a^ (1.31–1.53)	1.22^a^ (1.11–1.34)	1.53^a^ (1.43–1.63)	1.37^a^ (1.25–1.49)

AMO Tecnis group consists of ZCB00; Alcon AcrySof group consists of SA60AT and SN60WF IQ; B & L Akreos group consists of Adapt and MICS MI60; Lenstec group consists of Softec 1 and Softec HD; Rayner Flex group consists of C-Flex 970C and Superflex 920H lenses

^a^*p*-value <   0.05

The second largest observed impact was in relation to post-operative complications: at 3 years and 5 years, eyes with any post-operative complications were 3.03 (*P* < 0.001) and 4.76 (*P* < 0.001) more likely to experience Nd:YAG; the risk was even higher for PCO, where eyes with post-operative complications were 4.15 (*P* < 0.001) and 4.5 (*P* < 0.001) more likely to experience this at 3 and 5 years, respectively. In addition, the presence of copathologies recorded up to the data of cataract surgery had a impact on the risk of both PCO and Nd:YAG; at 5 years, eyes with any copathology were 1.22 (*P* < 0.001) more likely to receive Nd:YAG treatment, and 1.37 (*P* < 0.001) more likely to experience PCO. Similar trends were observed at 3 years post-cataract surgery.

Additional covariates that appear to increase the risk of Nd:YAG and PCO include younger age, female gender, having cataract surgery on both eyes (rather than one eye only), a lower IOL power at index, and better visual acuity (Table 3).

## Discussion

This study was an extension and post-hoc analysis of a previously published 3-year real-world evidence study [8] performed to collect 3-year and 5-year data on PCO and Nd:YAG incidence with each IOL group in this patient population. To evaluate the effect of IOL materials without the confounding factor of haptic design, this comparison included only single-piece IOLs (data from 47,754 eyes were included in the 3-year follow-up, and data from 20,763 eyes were included in the 5-year follow-up).

Choice of IOL can influence long-term outcomes. This study demonstrates that amongst all the single-piece IOL models implanted at the seven NHS sites, AcrySof IOLs demonstrated the lowest incidence of PCO and Nd:YAG capsulotomy at both 3 and 5 years. These findings remained statistically significant when adjusting for confounding variables.

These results are consistent with those from other long-term, retrospective, real-world studies that compared hydrophobic and hydrophilic IOLs with regards to Nd:YAG and PCO outcomes following cataract surgery [2, 6]. A 4-year study of German claims data found that hydrophobic IOLs were associated with significantly lower rates of PCO requiring Nd:YAG laser treatment and lower per-patient postoperative costs than hydrophilic IOLs [6]. Similarly, a retrospective study in Sweden (mean follow-up time, 41.5 months) found that hydrophobic IOLs had lower rates of Nd:YAG capsulotomy and PCO, and lower average costs for PCO treatment than hydrophilic IOLs [2]. It has been found that the acrylate material used in AcrySof IOLs shows different fibronectin binding compared with other hydrophobic materials; this may also offer a rationale for the lower PCO and subsequent Nd:YAG rates associated with AcrySof IOLs compared with other IOLs [21].

Edge design has also been shown to provide an important role in development of PCO and subsequently the risk of Nd:YAG capsulotomy [5] with previous research [22–28] demonstrating that IOLs with a square-edged optic profile are associated with less PCO than those with a round-edged profile. While all of the IOLs assessed in this study are marketed as having a square-edged profile, it could be the case that the degree of sharpness of the posterior optic edge may have some bearing on the variation in the PCO inhibiting properties displayed by different IOLs [29]. In addition, a study [30] reported that IOLs with a radius of curvature of <10.0 mm appear to have good PCO performance.

Our findings also demonstrate that besides IOLs, other factors including patient demographics, presence of other co-pathologies and post-op complications seem to influence long term PCO and Nd:YAG outcomes.

Factors that reduce the rates of PCO and Nd:YAG capsulotomy following cataract surgery are important for improving patient outcomes, as PCO involves loss of clear vision [21], and are likely to contribute to cost savings, as PCO and Nd:YAG represent considerable costs in the management of cataracts [1, 2, 6]. In addition to considering the cost of the Nd:YAG procedure itself, the prevention of PCO and subsequent Nd:YAG capsulotomies could reduce healthcare resource use required for PCO diagnosis, as well as any costs related to post-procedural monitoring, complications, and further treatments.

Strengths of this study include its longitudinal design and large sample size, which provided robust statistical power for comparative analyses; and use of Medisoft EMR data, a validated and widely accepted source of research data. Other important strengths include selection of NHS clinics in this study with completeness of the data collection and consistency of recording by using the Medisoft EMR, which enabled data pooling from multiple sites, and the appropriate statistical methods such as Bonferroni adjustment to account for multiplicity and adjusting for bias by conducting multivariate adjusted logistic regression analysis.

The Medisoft EMR database has comprehensive coverage of patients’ ophthalmic clinic related data. However, other relevant healthcare data (e.g. primary care, other secondary care) are not captured, thus reducing visibility of patients’ entire medical history a potential limitation and weakness of the study. Other limitations include loss to follow up and missing data (especially with regards to death), which are typical in observational database studies using secondary care data. Due to the lack of an indicator that a patient had de-registered from the hospital or moved to another area, it was assumed in this analysis that if a patient had cataract surgery before 2013, and no record of patient death was found, then that patient was assumed to have been followed for at least 3 years (5 years if the surgery was done between 2010 and 2011); this could lead to an overestimation of follow-up time, or underestimation of incidence of Nd:YAG and PCO. However, this was assumed to be evenly distributed between the different groups.

This study represents analysis conducted on a large dataset of real-world evidence regarding Nd:YAG and PCO incidence rates evaluated among patients with cataract surgery. Efforts to reduce the incidence of Nd:YAG and PCO following cataract surgery are likely to improve patient visual outcomes, satisfaction, and reduce healthcare resource costs.

In conclusion, this retrospective, real-world evidence study demonstrates that the choice of IOL implanted at the time of cataract surgery plays a considerable role in the risk of developing PCO and subsequent Nd:YAG capsulotomy treatment rates. When comparing all of the single-piece IOLs, AcrySof IOLs are associated with the lowest incidences of PCO and Nd:YAG capsulotomy over follow-up periods of 3 and 5 years post-cataract surgery. Further research is warranted to document the long-term (>5 years) benefits of different IOL materials and design on these outcomes.

### Summary

#### What was known before

- The risk of posterior capsule opacification post-cataract surgery is influenced by a number of factors, including edge design, IOL design, haptic design, and lens material
- Choice of IOL can influence long-term outcomes
- Previous studies suggest that more favourable outcomes have been shown for hydrophobic acrylic lenses compared with those made from other materials

#### What this study adds

- Our findings demonstrate that besides IOLs, other factors including patient demographics, presence of other co-pathologies, and post-op complications seem to influence long term PCO and Nd:YAG outcomes
- AcrySof IOLs were associated with significantly lower incidence of PCO requiring Nd:YAG treatment over periods of 3 and 5 years
- This study included a very large population of over 20,000 eyes undergoing cataract surgery, thus providing robust statistical analyses
